# Early Hemogram-Derived Inflammatory Indices Do Not Independently Predict Hospital Stay in Children with Human Bocavirus Detected by Polymerase Chain Reaction

**DOI:** 10.3390/children13101285

**Published:** 2026-09-22

**Authors:** Ali Kansu Tehçi, Mehmet Tolga Köle, Deniz Güven, İbrahim Kandemir, Öznur Karaca Vural, Saadet Paker Gülcan, Fuat Gezer, Emel Örün, Eyüp Sarı

**Affiliations:** 1 Department of Pediatrics, Ankara Etlik City Hospital, 06010 Ankara, Türkiye; mehmettolga.kole@saglik.gov.tr (M.T.K.); deniz.guven@sbu.edu.tr (D.G.); oznur.karaca1@saglik.gov.tr (Ö.K.V.); saadet.paker@saglik.gov.tr (S.P.G.); fuat.gezer@sbu.edu.tr (F.G.); emel.orun@sbu.edu.tr (E.Ö.); eyup.sari@sbu.edu.tr (E.S.); 2 Department of Pediatrics, Bahcelievler Medipol University Hospital, Istanbul Medipol University, 34196 Istanbul, Türkiye; ibrahim.kandemir@medipol.com.tr

**Keywords:** human bocavirus, systemic immune-inflammation index, neutrophil-to-lymphocyte ratio, platelet-to-lymphocyte ratio, inflammatory biomarkers, length of hospital stay, pediatric respiratory infections

## Abstract

**Highlights:**

**What are the main findings?**

- Early SII, NLR, PLR, and NPAR were inversely associated with length of hospital stay but did not independently predict hospitalization duration after adjustment for clinical covariates.
- Neurological disease was associated with longer hospitalization, whereas higher neutrophil percentage and tachycardia were independently associated with shorter expected length of hospital stay.

**What are the implications of the main findings?**

- Hemogram-derived inflammatory indices may provide adjunctive clinical information but should not be used as stand-alone prognostic markers in children with PCR-detected HBoV.
- Length of hospital stay should be interpreted cautiously as a severity endpoint because it is influenced by comorbidity, clinical management, discharge practices, and other process-related factors.

**Abstract:**

Background/Objectives: Human bocavirus (HBoV) is frequently detected by polymerase chain reaction (PCR) in respiratory samples from children with acute respiratory tract disease, but prolonged viral DNA persistence and frequent co-detection complicate interpretation of its clinical significance. We evaluated whether early hemogram-derived inflammatory indices are associated with clinical outcomes in hospitalized children with PCR-detected HBoV. Methods: This single-center retrospective cohort included 170 hospitalized children aged 1 month to 18 years with HBoV detected by PCR in nasopharyngeal samples between July 2022 and June 2025. Data obtained within the first 24 h of hospitalization were analyzed. Systemic immune-inflammation index (SII), neutrophil-to-lymphocyte ratio (NLR), platelet-to-lymphocyte ratio (PLR), and neutrophil percentage-to-albumin ratio (NPAR) were calculated. Associations with clinical outcomes were assessed using correlation analyses, and multivariable generalized linear modeling was used to evaluate factors associated with length of hospital stay. Results: Median age was 18 months (IQR, 11–33), and microbiological co-detection was present in 60.4%. Oxygen therapy was required in 82.8%, high-flow nasal cannula support in 22.0%, and PICU admission in 5.4%. All four indices were inversely correlated with length of hospital stay and blood gas bicarbonate levels. However, none independently predicted length of stay after adjustment. Neurological disease, neutrophil percentage, and tachycardia remained independently associated with length of stay; neurological disease was associated with a 1.56-fold longer expected hospitalization, whereas higher neutrophil percentage and tachycardia were associated with shorter expected hospitalization. Conclusions: Early hemogram-derived inflammatory indices were associated with clinical course but did not independently predict hospitalization duration in children with PCR-detected HBoV. Their prognostic value should be interpreted cautiously, particularly when length of stay is used as a process-sensitive outcome, and the observed associations should not be considered HBoV-specific.

## 1. Introduction

Since its identification in 2005, Human Bocavirus (HBoV) has been frequently detected in respiratory samples from children with acute respiratory tract infections; however, its causal role remains controversial. Acute respiratory tract infections and community-acquired pneumonia are among the leading causes of hospitalization, particularly in infancy and early childhood. In previous hospital-based series, HBoV—particularly HBoV1, the subtype most commonly linked to respiratory disease—has been detected in approximately 2% to 10% of pediatric respiratory samples, suggesting that the clinical relevance of HBoV detection warrants further investigation [1,2,3,4]. However, the clinical interpretation of HBoV PCR positivity remains difficult because prolonged persistence of viral DNA in nasopharyngeal samples may yield PCR positivity without indicating active acute infection, while frequent co-detection with other respiratory pathogens further complicates causal inference [2,3,5]. Accordingly, establishing pathogenicity more reliably may require quantitative viral load assessment, markers of active replication, and serological confirmation [6,7,8].

In hospitalized children with HBoV detected in respiratory samples, the clinical presentation spans a broad spectrum ranging from bronchiolitis and wheezing episodes to pneumonia; severe outcomes, including oxygen requirement, need for advanced respiratory support, and admission to the pediatric intensive care unit (PICU), may be more pronounced in certain subgroups [9,10]. In addition, microbiological co-detection has been shown in some studies to be associated with prolonged hospitalization and an increased need for respiratory support [9,11,12]. This clinical heterogeneity underscores the growing need for rapid, readily applicable, and cost-effective biomarkers that can facilitate early prediction of clinically meaningful outcomes, such as length of hospital stay and PICU requirement, in HBoV-positive cases.

Clinical deterioration in pneumonia and severe lower respiratory tract infections is largely driven by the magnitude of the host inflammatory response. Accordingly, hemogram-derived indices that can be readily calculated from complete blood count parameters, including the neutrophil-to-lymphocyte ratio (NLR), monocyte-to-lymphocyte ratio (MLR), platelet-to-lymphocyte ratio (PLR), and the systemic immune-inflammation index (SII), have garnered increasing interest in recent years for clinical risk stratification. Available evidence suggests that SII may contribute to severity discrimination in bronchiolitis and lower respiratory tract infections; however, its predictive performance appears to vary depending on the underlying etiological diagnosis and the clinical outcomes assessed [13,14,15]. Evidence from bronchiolitis and pediatric pneumonia cohorts suggests that inflammatory indices such as NLR, MLR, and SII, as well as lymphocyte-based markers, may help identify more severe clinical phenotypes [16,17,18,19]. Emerging data have shown that HBoV infection may be accompanied by marked elevations in inflammatory markers, suggesting a potential link between inflammatory activation and clinical severity [20].

To our knowledge, despite frequent reporting of NLR and PLR in broader viral respiratory cohorts, outcome-oriented evidence on early hemogram-derived inflammatory indices in HBoV-positive hospitalized children remains limited, particularly with respect to the combined evaluation of SII, NLR, PLR, and neutrophil percentage-to-albumin ratio (NPAR). Therefore, we aimed to evaluate the association between early hemogram-derived indices (SII, NLR, PLR, and NPAR) and key clinical outcomes, with a particular focus on length of hospital stay and PICU requirement, in children hospitalized with HBoV detected by PCR in nasopharyngeal respiratory samples. We found that these indices were associated with markers of clinical course but did not independently predict length of hospital stay after adjustment for clinical covariates, whereas neurological disease, neutrophil percentage, and tachycardia remained independently associated with length of stay.

## 2. Materials and Methods

### 2.1. Study Design and Population

This single-center retrospective cohort study was conducted at a tertiary-care hospital in Ankara, Türkiye. Hospitalized children aged 1 month to 18 years in whom HBoV was detected by PCR in nasopharyngeal respiratory samples between 1 July 2022 and 30 June 2025 were included. Patients with insufficient data to establish study eligibility were excluded. Missing individual clinical and laboratory measurements were handled using an available-case approach. The study protocol was approved by the Ethics Committee of Ankara Etlik City Hospital (AEŞH-BADEK1-2025-374; 9 July 2025) and was conducted in accordance with the Declaration of Helsinki.

### 2.2. Data Collection and Clinical Definitions

Demographic, clinical, and laboratory data were retrospectively retrieved from the hospital’s electronic medical records. Recorded variables included age, sex, comorbidities, presenting clinical features, treatment and follow-up data during hospitalization, and co-detection status. Patients were followed from hospital admission until discharge; no post-discharge follow-up data were available. Laboratory parameters and chest radiographic findings, when available, were obtained from tests performed within the first 24 h of hospitalization. For blood culture findings, data required for standardized adjudication of true bloodstream infection versus contamination were not consistently available in the retrospective records. In this study, the term “early” refers to laboratory values obtained within the first 24 h of hospitalization, rather than the time from symptom onset. Reliable information on symptom onset and pre-admission symptom duration was not consistently available in the retrospective records; therefore, day of illness at blood sampling could not be included as a covariate in the adjusted analyses.

Hemogram-derived inflammatory indices were calculated as follows: NLR as neutrophil count divided by lymphocyte count, PLR as platelet count divided by lymphocyte count, SII as platelet count × neutrophil count/lymphocyte count, and NPAR as neutrophil percentage divided by serum albumin.

Lower respiratory tract infection was defined as a clinical syndrome characterized by lower respiratory symptoms accompanied by fever, with evidence of parenchymal involvement on physical examination and/or chest radiography [21]. Admission chest radiograph findings were classified using PERCH-style chest radiograph conclusion categories as normal, only consolidation or pleural effusion without other infiltrate, other infiltrate without consolidation, both consolidation and other infiltrate, or uninterpretable [22]. Patients without an available admission chest radiograph were recorded separately. In cases of clinical deterioration during hospitalization or when otherwise clinically indicated, advanced imaging modalities, including chest computed tomography, were performed.

### 2.3. Microbiological Analysis

Nasopharyngeal specimens were collected using Dacron or polyester swabs and transported in Bio-Speedy^®^ vNAT^®^ Viral Transfer Tubes (Cat. No. BS-NA-513-100; Bioeksen R&D Technologies, Istanbul, Türkiye). Specimen analysis was performed using the Bio-Speedy^®^ Respiratory Tract RT-qPCR MX-24S Panel kit (Bioeksen R&D Technologies, Istanbul, Türkiye), which operates on a one-step reverse transcription and real-time polymerase chain reaction (RT-qPCR) principle to detect pathogen-specific RNA/DNA targets. All amplifications were carried out on a CFX96 Real-Time PCR Detection System (Bio-Rad Laboratories, Inc., Hercules, CA, USA).

The panel screened for the following viral pathogens: SARS-CoV-2, influenza A/B, human coronaviruses (229E, OC43, NL63, HKU1), parainfluenza virus types 1–4, human metapneumovirus, respiratory syncytial virus A/B, human enterovirus, adenovirus, HBoV, human parechovirus, and human rhinovirus. The panel reported HBoV as a single qualitative target, and genotype-specific results were not available in the study dataset. Bacterial targets included Legionella pneumophila, Mycoplasma pneumoniae, Chlamydophila pneumoniae, Haemophilus influenzae, Bordetella pertussis, and Streptococcus pneumoniae.

### 2.4. Statistical Analysis

Data are expressed as number (percentage), median (interquartile range [IQR]), or mean ± standard deviation (SD), as appropriate. The distribution of continuous variables was assessed using the Kolmogorov–Smirnov test, skewness and kurtosis measures, and Q–Q plots. Continuous variables were compared between groups using Student’s *t* test or the Mann–Whitney U test, as appropriate. Subgroup comparisons were performed according to recorded blood culture growth status to assess differences in hemogram-derived inflammatory indices and clinical outcomes.

Correlation analyses were performed using Spearman’s rank correlation coefficient, with pairwise complete observations used for analyses involving variables with missing data. To control the false discovery rate arising from multiple correlation analyses, the *p* values from all 70 Spearman correlation tests were jointly adjusted using the Benjamini–Hochberg procedure. Both unadjusted *p* values and false discovery rate (FDR)-adjusted q values are reported, with q < 0.05 considered statistically significant. These correlation analyses were considered exploratory and hypothesis-generating. To identify factors independently associated with the outcome, a generalized linear model was constructed using forward selection from variables identified in univariate analyses and clinically relevant covariates. Multicollinearity was assessed during model development. Because NLR, PLR, SII, and NPAR share overlapping hematologic components and are intrinsically correlated, they were not entered simultaneously into the same multivariable model. Instead, four separate multivariable models were constructed, each including one hemogram-derived inflammatory index together with the same clinically relevant covariates, including tachycardia, neutrophil percentage, neurological disease, inborn errors of metabolism, and cardiac disease. A two-tailed *p* value of <0.05 was considered statistically significant. Statistical analyses were performed using JAMOVI software, version 2.6.44.

## 3. Results

### 3.1. Demographic and Clinical Characteristics

A total of 170 hospitalized children with HBoV detected by PCR in nasopharyngeal respiratory samples were included in the study. Of these, 106 (62.4%) were male, and the median age was 18 months (IQR, 11–33 months). Admissions showed seasonal variation, with the highest proportion occurring during winter (34.1%). At least one comorbid condition was present in 53 patients (31.2%), most commonly neurologic (14.7%) and pulmonary disorders (11.2%). The leading presenting symptoms were cough (77.6%), respiratory distress (58.0%), and fever (49.4%). On physical examination, rales/rhonchi and retractions were observed in 67.1% and 61.2% of patients, respectively, whereas oxygen desaturation was documented in 37.9%. Microbiological co-detection was identified in 102 patients (60.4%), most frequently involving Streptococcus pneumoniae (32.4%) and Haemophilus influenzae (24.1%). Abnormal radiographic findings were identified in 97 patients (57.1%). Microbial growth in blood cultures was documented in 19 patients (11.2%). Isolates included Micrococcus luteus (n = 6), Staphylococcus epidermidis (n = 4), Staphylococcus hominis (n = 2), and one isolate each of Streptococcus pneumoniae, Staphylococcus aureus, Streptococcus mitis/oralis, Actinomyces species, Streptococcus parasanguinis, and Candida albicans. One polymicrobial blood culture yielded Enterococcus faecalis and Pluralibacter gergoviae. Oxygen therapy was required in 82.8% of patients, HFNC in 22.0%, and PICU admission in 5.4%; the mean PICU stay was 4.8 ± 2.5 days, and the median hospital stay was 6 days (IQR, 5–9 days) (Table 1).

### 3.2. Laboratory Findings and Radiographic Subgroup Analyses

Baseline laboratory findings are summarized in Table 2. The median white blood cell count was 12.08 ×10^3^/µL, the median neutrophil percentage was 62.85%, and the median CRP level was 9.66 mg/L. The median blood gas pH was 7.396, and the median bicarbonate level was 20.4 mmol/L. Hemogram-derived inflammatory indices showed wide interindividual variability, with median values of 1.42 for NPAR, 841.9 for SII, 2.23 for NLR, and 124.4 for PLR.

In subgroup analyses, none of the hemogram-derived inflammatory indices differed significantly according to chest radiograph findings. When each radiographic category was compared with all remaining patients, no significant differences were observed in patients with normal radiographs (NPAR, *p* = 0.436; SII, *p* = 0.986; NLR, *p* = 0.602; PLR, *p* = 0.981), consolidation and/or pleural effusion without other infiltrates (NPAR, *p* = 0.781; SII, *p* = 0.515; NLR, *p* = 0.599; PLR, *p* = 0.170), other infiltrates without consolidation (NPAR, *p* = 0.231; SII, *p* = 0.614; NLR, *p* = 0.389; PLR, *p* = 0.931), or concomitant consolidation and other infiltrates (NPAR, *p* = 0.446; SII, *p* = 0.342; NLR, *p* = 0.344; PLR, *p* = 0.255).

### 3.3. Correlation and Blood Culture Subgroup Analyses

Correlations between inflammatory indices and clinical parameters are presented in Table 3. All four indices were significantly and inversely correlated with length of hospital stay, including NPAR (*p* < 0.01), SII (*p* < 0.01), NLR (*p* < 0.01), and PLR (*p* < 0.01). In addition, all indices showed inverse correlations with blood gas bicarbonate levels. Among the individual indices, SII showed a nominal inverse association with oxygen saturation (*p* < 0.05), whereas NLR showed a nominal inverse association with HFNC duration (*p* < 0.05); however, neither association remained statistically significant after FDR correction (q > 0.05). SII, NLR, and PLR also showed weak inverse associations with antibiotic treatment duration.

In subgroup analyses according to recorded blood culture growth status, no statistically significant differences were observed in NPAR (*p* = 0.213), SII (*p* = 0.732), NLR (*p* = 0.426), PLR (*p* = 0.490), or length of hospital stay (*p* = 0.398). Similarly, WBC count (*p* = 0.478), neutrophil count (*p* = 0.764), lymphocyte count (*p* = 0.344), procalcitonin (*p* = 0.275), and CRP (*p* = 0.051) did not differ significantly according to recorded blood culture growth status.

### 3.4. Factors Associated with Length of Hospital Stay

Spearman’s rank correlation analysis showed that length of hospital stay was inversely associated with neutrophil percentage, glucose, and albumin, whereas positive correlations were observed with lymphocyte percentage, potassium, pCO_2_, and HCO_3_^−^. Creatinine, alanine aminotransferase (ALT), and calcium showed nominal associations with length of hospital stay in the unadjusted analyses (all *p* < 0.05); however, these associations did not remain statistically significant after FDR correction (q > 0.05). Associations between length of hospital stay and hemogram-derived inflammatory indices are presented separately in Table 3. No significant associations were identified for the remaining baseline clinical and laboratory variables (Table 4). Tachycardia (ρ = −0.174, *p* < 0.01) was associated with length of hospital stay, whereas oxygen desaturation (ρ = 0.144) and microbiological co-detection (ρ = 0.024) were not. In exploratory subtype analyses, neurological disease (n = 25, ρ = 0.286, *p* < 0.001), inborn errors of metabolism (n = 7, ρ = 0.192, *p* < 0.01), and cardiac disease (n = 11, ρ = 0.167, *p* < 0.05) were positively associated with length of hospital stay.

Because lymphocyte and neutrophil percentages were highly collinear, only neutrophil percentage was retained in the multivariable analyses. NPAR, SII, NLR, and PLR were each evaluated in separate multivariable models together with tachycardia, neutrophil percentage, neurological disease, inborn errors of metabolism, and cardiac disease. None of the four hemogram-derived inflammatory indices remained statistically significant after FDR correction (Supplementary Table S1).

Tachycardia, neurological disease, and neutrophil percentage remained independently associated with length of hospital stay. Neurological disease was associated with a 1.56-fold longer expected hospital stay (95% CI, 1.03–2.37; *p* = 0.043). Each 1% increase in neutrophil percentage was associated with a 0.984-fold change in expected length of hospital stay (95% CI, 0.976–0.992; *p* < 0.001), whereas tachycardia was associated with a 0.57-fold change (95% CI, 0.36–0.91; *p* = 0.012). The final model had an adjusted R^2^ of 0.242. The estimated marginal means are presented in Figure 1.

## 4. Discussion

Although HBoV is frequently detected in respiratory samples from children with acute respiratory tract disease, it has long been considered a potential “bystander” virus because of high rates of co-detection and prolonged viral shedding or persistence. However, accumulating clinical evidence demonstrating that severe lower respiratory tract infections may occur even in cases with mono-infection suggests a genuine pathogenic potential of HBoV [23]. In this single-center retrospective study, we observed a substantial clinical burden among 170 hospitalized children in whom HBoV was detected by PCR in nasopharyngeal respiratory samples. The majority of cases occurred in infancy and early childhood, with a median age of 18 months. The increase in admissions during the winter months is consistent with multi-season hospital-based series reporting higher detection rates of HBoV, particularly HBoV1, during colder seasons [3,24,25]. On the other hand, reports from different geographic regions describing summer–autumn predominance or a more homogeneous year-round circulation suggest that the epidemiology of HBoV may be influenced by contextual factors such as regional climate conditions and healthcare utilization patterns [26]. In addition, public health interventions implemented during the COVID-19 pandemic have been shown to alter the circulation patterns of respiratory viruses and to contribute to temporal fluctuations in HBoV prevalence, underscoring the importance of interpreting seasonality findings within the relevant epidemiological context [27].

One of the key findings of our study is the high rate of co-detection (60.4%). This observation is consistent with previous literature showing that HBoV, particularly HBoV1 in subtype-specific studies, is frequently identified alongside other respiratory pathogens in children with acute respiratory tract disease [2,3,28]. However, the impact of co-detection on clinical outcomes appears to be variable across studies: while some series have reported associations with prolonged hospitalization and increased need for intensive care or advanced respiratory support [9,11], others have not demonstrated significant differences [24]. This heterogeneity may be explained, at least in part, by the fact that PCR positivity in nasopharyngeal samples does not always reflect acute pathogenicity, given the potential for prolonged viral shedding/persistence and the possibility of bystander detection [29]. Accordingly, it has been emphasized that complementary approaches, such as assessment of Ct values/viral load, markers of active replication (e.g., capsid mRNA), and/or serological confirmation, may enhance etiological attribution in HBoV-positive cases [4,8,30]. Notably, evidence showing that severe lower respiratory tract infection can develop in the presence of high viral load even in the absence of other detected pathogens further supports the notion that HBoV1 may act as a true pathogen under appropriate clinical conditions [23,31].

From a clinical perspective, the requirement for oxygen therapy in 82.8% of patients and HFNC use in 22.0% indicate a considerable clinical burden in this hospitalized cohort. This pattern is consistent with reports indicating that hypoxemia and the need for respiratory support are common among hospitalized pediatric HBoV cases [9,28]. In contrast, pediatric intensive care unit (PICU) admission in our cohort was limited to 5.4% (mean PICU length of stay, 4.8 days), approximating the lower bound of rates reported in the literature. For instance, a cohort of 165 cases reported a PICU admission rate of 7.3% [9], whereas a larger series documented PICU monitoring in 16.26% of children [2]. Although the relatively broad availability of ward-based HFNC at our center may have enabled earlier optimization of respiratory support in selected patients and thereby reduced the need for escalation to intensive care, the retrospective design and potential differences in case mix preclude causal inference. In this context, variability in PICU requirement should be interpreted with consideration of center-specific practices and heterogeneity in patient profiles.

The primary novel contribution of our study is the systematic evaluation of the associations between early hemogram-derived inflammatory indices calculated shortly after presentation and indicators of clinical course in children hospitalized with PCR-detected HBoV. In this context, the inverse correlations of NPAR, SII, NLR, and PLR with blood gas bicarbonate levels suggest that the systemic inflammatory response may increase in parallel with respiratory and metabolic burden. However, chest radiograph categories did not show meaningful differences in these indices, indicating that early hemogram-derived inflammatory indices may not reliably discriminate specific radiographic patterns in this cohort. These findings may be interpreted as supporting, within the specific context of HBoV infection, prior evidence indicating that indices such as SII and NLR can be associated with severity phenotypes in pediatric lower respiratory tract infections [15,16]. Moreover, pediatric pneumonia cohorts showing that PLR and albumin-based ratios may contribute to discriminating more severe clinical phenotypes further reinforce the link between the inflammation–nutrition axis and clinical outcomes, highlighting the potential role of hemogram-derived indices in risk stratification [32,33]. Therefore, the observed associations should not be interpreted as HBoV-specific inflammatory signatures, but rather as markers of clinical and inflammatory burden among children hospitalized with PCR-detected HBoV.

However, the most striking finding of our study was the inverse relationship between hemogram-derived inflammatory indices and length of hospital stay, with significant negative correlations observed for NPAR, SII, NLR, and PLR. This pattern suggests that, in pediatric respiratory infections, length of stay may not represent a direct or “pure” proxy of biological severity; rather, it may be a process-sensitive outcome shaped by multiple determinants, including discharge decision-making, care pathways, comorbidity burden, and center-specific practices. Consistent with this interpretation, the multivariable analysis showed that neurological disease was independently associated with longer hospitalization, whereas higher neutrophil percentage and the presence of tachycardia were independently associated with shorter expected length of hospital stay. These findings further support the view that length of hospital stay is influenced by multiple patient-level and care-related factors and should not be interpreted as a direct proxy of inflammatory or biological disease severity. The inverse associations observed for neutrophil percentage and tachycardia should therefore be interpreted cautiously and may reflect residual confounding or other process-related determinants of hospitalization. Previous work has suggested that HBoV1 may modulate local immune responses in persistent tonsillar infection [34]; however, the relevance of this mechanism to acute hospitalized respiratory disease and to the inverse associations observed in our cohort remains uncertain.

In this respect, the observed inverse association should be considered not as a direct contradiction to the commonly reported parallel trend between elevated inflammatory indices and more severe phenotypes, but rather as a hypothesis-generating signal that highlights the process-sensitive nature of length of stay and the potential for residual confounding in HBoV-positive hospitalized populations. Indeed, beyond biological disease severity, length of stay is a multidimensional indicator that may be influenced by management strategies, discharge criteria, and patient-specific characteristics. Future studies incorporating stratified analyses by age group, comorbidity status, co-detection patterns, and clinical severity markers such as oxygen or HFNC requirements, and, where feasible, including serial measurements of inflammatory indices, will be essential to clarify whether the observed relationship reflects underlying biological mechanisms or predominantly process-sensitive dynamics.

Key strengths of our study include its reliance on real-world data collected over a three-year period, the concurrent evaluation of multiple clinically relevant outcomes such as length of hospital stay, requirement for oxygen support, and PICU admission, and its focus on hemogram-derived inflammatory indices calculated from complete blood count parameters obtained within the first 24 h of presentation, which can be readily integrated into routine clinical practice.

Nevertheless, the retrospective design and single-center sampling limit the generalizability of our findings. Because inflammatory indices were assessed at a single time point within the first 24 h of hospitalization, temporal directionality cannot be established, and reverse causation and confounding by indication cannot be excluded. Symptom-onset timing was not consistently available, precluding adjustment for day of illness at blood sampling and leaving the analyses susceptible to residual confounding by illness stage. Accordingly, the inverse associations with length of stay should not be interpreted as evidence of a causal relationship between the inflammatory profile and faster clinical recovery. The relatively small number of PICU cases may have reduced statistical power, particularly for analyses of severe clinical outcomes. Although exploratory analyses were performed for selected comorbidity subtypes, the primary comorbidity variable was analyzed as a composite binary measure encompassing heterogeneous underlying conditions. Therefore, subtype-specific associations should be interpreted cautiously, particularly given the small sample sizes within individual comorbidity categories. The absence of a symptomatic HBoV-negative or alternative-pathogen control group limits causal interpretation and prevents determination of whether the observed inflammatory patterns are specific to HBoV detection or reflect the broader inflammatory burden of pediatric lower respiratory tract infection. In addition, the absence of data that could help distinguish active infection from persistent viral DNA detection, such as Ct values or viral load, capsid mRNA, or serological confirmation, represents an important limitation for etiological interpretation. A further limitation is that the PCR assay did not differentiate among HBoV genotypes 1–4; therefore, genotype-specific attribution, particularly to HBoV1, was not possible. Cytokine markers such as IL-6 and TNF-α were not routinely available and therefore could not be evaluated. Pneumococcal and influenza vaccination records were not consistently available in the retrospective dataset and could not be analyzed. Blood culture findings should also be interpreted cautiously because the available retrospective data did not permit standardized adjudication of true bloodstream infection versus contamination. Accordingly, analyses based on blood culture growth status reflect recorded microbial growth rather than adjudicated bloodstream infection. Moreover, because some bacterial targets detected by molecular panels in nasopharyngeal specimens may reflect colonization rather than true infection, the definition of “microbiological co-detection” should be interpreted cautiously in conjunction with clinical and radiological findings. Also, although the Benjamini–Hochberg procedure was applied to control the false discovery rate across the correlation analyses, the large number of comparisons and the intrinsic correlations among the hemogram-derived indices should still be considered when interpreting these exploratory findings. Therefore, the observed associations should be regarded as hypothesis-generating and require confirmation in independent cohorts.

## 5. Conclusions

In conclusion, children hospitalized with PCR-detected HBoV showed a heterogeneous clinical course and a high rate of microbiological co-detection. Oxygen therapy and HFNC requirement indicate a considerable clinical burden, whereas PICU utilization may be influenced by center-specific practices and differences in case mix. Early hemogram-derived inflammatory indices, including SII, NLR, PLR, and NPAR, may have adjunctive value for the assessment of clinical course; however, their associations should be interpreted cautiously, particularly when process-sensitive outcomes such as length of hospital stay are used. In the absence of an HBoV-negative or alternative-pathogen control group, the observed associations should be interpreted cautiously with regard to HBoV specificity and may partly reflect the broader inflammatory burden of pediatric lower respiratory tract infection. Prospective multicenter studies are needed to determine whether these indices provide independent and clinically meaningful prognostic information in children with PCR-detected HBoV. In addition, approaches that improve virological specificity, such as viral load assessment, mRNA-based markers, or serological confirmation, together with the use of standardized severity endpoints, may help clarify their clinical utility.

## Figures and Tables

**Figure 1:**
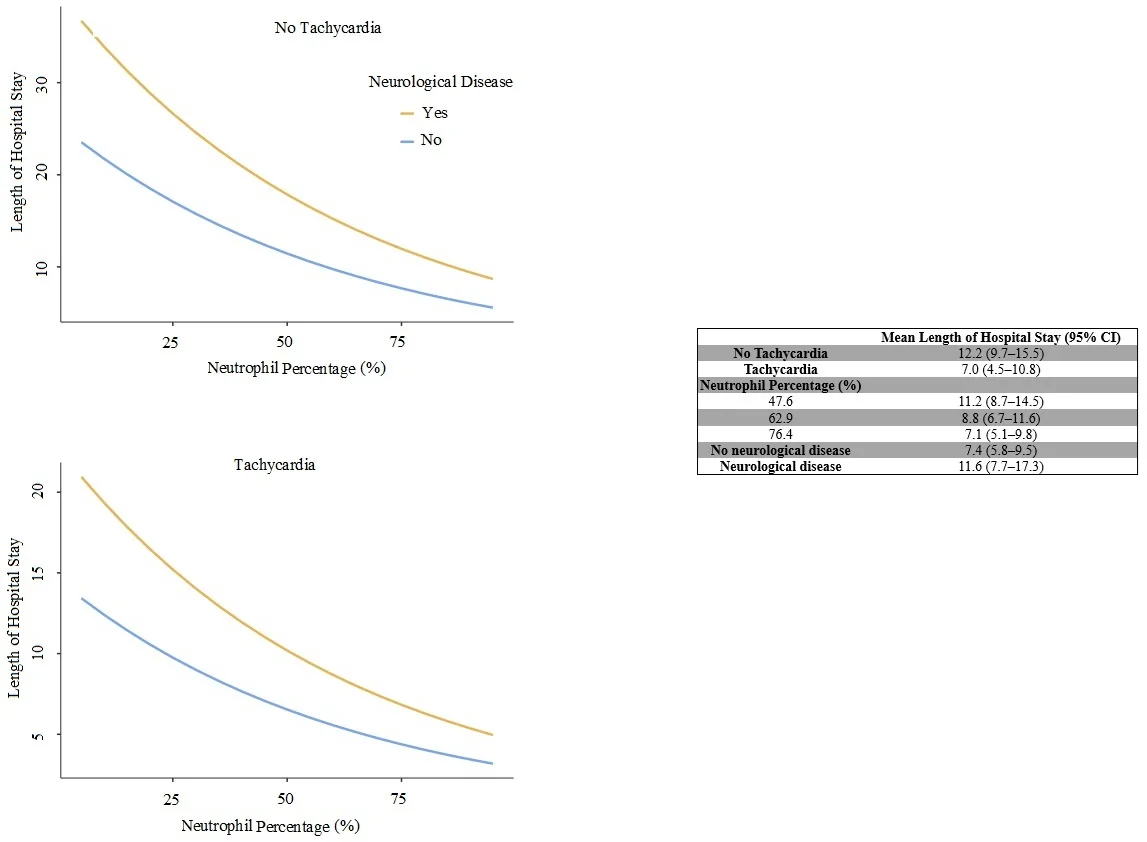
Model-based predicted length of hospital stay according to neutrophil percentage, tachycardia, and the presence of neurological disease. Estimated marginal means are presented with 95% confidence intervals.

**Table 1:** Baseline demographic and clinical characteristics of the patients.

Variable	N	n (%)
**Sex**		
Male	170	106 (62.4)
Female	170	64 (37.6)
Age, months (median, IQR)	170	18 (11–33)
**Any comorbidity**	170	53 (31.2)
Lung disease	170	19 (11.2)
Neurologic disease	170	25 (14.7)
Cardiac disease	170	11 (6.5)
Inborn errors of metabolism	170	7 (4.1)
Other comorbidities	170	42 (24.7)
**Initial symptoms**		
Cough	170	132 (77.6)
High fever	170	84 (49.4)
Nasal congestion	170	43 (25.3)
Respiratory distress	169	98 (58.0)
Other symptoms	170	40 (23.5)
**Physical examination findings**		
Rales/rhonchi	170	114 (67.1)
Retractions	170	104 (61.2)
Nasal flaring	170	25 (14.7)
Tachypnea	170	100 (58.8)
Tachycardia	170	48 (28.2)
Oxygen desaturation	140	53 (37.9)
**Co-detection (any)**	169	102 (60.4)
Streptococcus pneumoniae	170	55 (32.4)
Mycoplasma pneumoniae	170	5 (2.9)
Haemophilus influenzae	170	41 (24.1)
Rhinovirus	170	17 (10.0)
Influenza virus	170	20 (11.8)
Human metapneumovirus	170	2 (1.2)
Parainfluenza virus	170	2 (1.2)
SARS-CoV-2	170	4 (2.4)
Blood culture with microbial growth	170	19 (11.2)
**Chest radiograph category**		
—Normal	170	66 (38.8)
—Only consolidation or pleural effusion without other infiltrate	170	13 (7.6)
—Other infiltrate without consolidation	170	65 (38.2)
—Both consolidation and other infiltrate	170	19 (11.2)
—Uninterpretable	170	2 (1.2)
—No available chest radiograph	170	5 (2.9)
**Treatment modalities**		
Steroid therapy	170	83 (48.8)
Magnesium sulfate	170	37 (21.8)
Oxygen therapy	169	140 (82.8)
High-flow nasal cannula	168	37 (22.0)
Non-invasive mechanical ventilation	168	2 (1.2)
Invasive mechanical ventilation	168	2 (1.2)
**Clinical course and outcomes**		
PICU admission	168	9 (5.4)
Length of PICU stay, days (mean ± SD)	9	4.8 ± 2.5
Length of hospital stay, days (median, IQR)	168	6 (5–9)

Data are presented as n (%) unless otherwise stated. N represents the number of patients with available data for the corresponding variable and may therefore vary because of missing documentation. Continuous variables are presented as median (interquartile range) or mean ± standard deviation, as appropriate. Percentages were calculated using the corresponding available-case denominator. PICU, pediatric intensive care unit; SD, standard deviation; IQR, interquartile range. Microbial growth in blood cultures was recorded from the medical record and was not adjudicated as true bloodstream infection versus contamination. Chest radiograph categories were based on PERCH-style conclusion categories; patients without an available admission chest radiograph were recorded separately.

**Table 2:** Laboratory parameters of the study population.

Parameter	N	Median (25th–75th Percentile)
WBC (×10^3^/µL)	170	12.08 (9.24–16.01)
Hemoglobin (g/dL)	170	11.65 (10.63–12.30)
Platelet (×10^3^/mm^3^)	170	375.5 (282.5–475.8)
Neutrophil (×10^3^/µL)	170	7.04 (4.15–10.58)
Neutrophil percentage (%)	170	62.85 (46.93–76.63)
Lymphocyte (×10^3^/µL)	170	2.99 (1.98–5.12)
Lymphocyte percentage (%)	170	28.40 (17.20–40.58)
Albumin (g/L)	169	43.7 (41.7–46.1)
CRP (mg/L)	167	9.66 (2.76–31.98)
Blood gas pH	137	7.396 (7.361–7.424)
pCO_2_ (mmHg)	135	33.7 (30.35–38.2)
HCO_3_^−^ (mmol/L)	137	20.4 (18.7–21.9)
Procalcitonin (ng/mL)	20	0.12 (0.10–0.30)
NPAR	169	1.42 (1.08–1.74)
SII	170	841.9 (380.6–1633.6)
NLR	170	2.23 (1.07–4.37)
PLR	170	124.4 (72.8–187.5)

Values are presented as median (25th–75th percentile). The sample size (n) indicates the number of patients with valid data available for each parameter. Analyses were performed using available cases; therefore, sample sizes varied across laboratory parameters. WBC, white blood cell count; CRP, C-reactive protein; NLR, neutrophil-to-lymphocyte ratio; PLR, platelet-to-lymphocyte ratio; SII, systemic immune-inflammation index; NPAR, neutrophil percentage-to-albumin ratio.

**Table 3:** Correlations between inflammatory indices and clinical parameters.

Clinical Parameter	NPAR, ρ (n)	SII, ρ (n)	NLR, ρ (n)	PLR, ρ (n)
Antibiotic treatment duration	−0.132 (155)	−0.180 * (156)	−0.164 * (156)	−0.170 * (156)
Oxygen therapy duration	−0.113 (137)	−0.114 (137)	−0.124 (137)	−0.156 (137)
HFNC duration	−0.271 (36)	−0.284 (36)	−0.396 * (36)	−0.259 (36)
Length of hospital stay	−0.224 ** (167)	−0.246 ** (168)	−0.234 ** (168)	−0.239 ** (168)
PICU length of stay	0.260 (9)	0.414 (9)	0.518 (9)	0.104 (9)
Body temperature	0.091 (128)	0.045 (129)	0.084 (129)	−0.074 (129)
Heart rate	−0.042 (115)	−0.046 (115)	−0.083 (115)	−0.111 (115)
Oxygen saturation	−0.144 (138)	−0.197 * (138)	−0.161 (138)	−0.092 (138)
Blood gas pH	0.092 (137)	0.042 (137)	0.042 (137)	0.102 (137)
Blood gas HCO_3_^−^	−0.271 ** (137)	−0.339 *** (137)	−0.333 *** (137)	−0.349 *** (137)
Blood gas lactate	−0.045 (137)	−0.007 (137)	−0.048 (137)	−0.006 (137)
CRP	0.259 *** (166)	0.108 (167)	0.208 ** (167)	−0.018 (167)

Values are presented as Spearman’s rank correlation coefficients (ρ), with the number of pairwise complete observations shown in parentheses. Sample sizes varied across variable pairs because of missing measurements. NPAR, neutrophil percentage-to-albumin ratio; SII, systemic immune-inflammation index; NLR, neutrophil-to-lymphocyte ratio; PLR, platelet-to-lymphocyte ratio; HFNC, high-flow nasal cannula; PICU, pediatric intensive care unit; CRP, C-reactive protein. * *p* < 0.05, ** *p* < 0.01,*** *p* < 0.001.

**Table 4:** Correlations between length of hospital stay and baseline clinical and laboratory parameters.

Variable	N	Spearman’s ρ
Age (months)	168	−0.033
White blood cell count (×10^3^/µL)	168	−0.001
Hemoglobin (g/dL)	168	−0.072
Platelet count (×10^3^/mm^3^)	168	−0.069
Neutrophil ratio (%)	168	−0.273 ***
Lymphocyte ratio (%)	168	0.242 **
Sodium (mmol/L)	166	−0.029
Potassium (mmol/L)	167	0.293 ***
Creatinine (mg/dL)	150	−0.176 *
Urea (mg/dL)	167	−0.086
Uric acid (mg/dL)	166	−0.119
Alanine aminotransferase (U/L)	164	0.178 *
Aspartate aminotransferase (U/L)	164	0.111
Glucose (mg/dL)	166	−0.329 ***
Calcium (mg/dL)	167	−0.176 *
Albumin (g/L)	167	−0.256 ***
C-reactive protein (mg/L)	165	−0.053
Blood gas pH	136	−0.103
pCO_2_ (mmHg)	134	0.268 **
HCO_3_^−^ (mmol/L)	136	0.269 **
Blood gas lactate (mmol/L)	136	−0.147
Procalcitonin (ng/mL)	19	−0.274

Values represent Spearman’s rank correlation coefficients (ρ). The sample size (n) indicates the number of pairwise complete observations available for each analysis. Sample sizes varied across variables because of missing measurements. * *p* < 0.05, ** *p* < 0.01,*** *p* < 0.001.

## Data Availability

The data that support the findings of this study are available from the corresponding author upon reasonable request. The data are not publicly available due to patient privacy and ethical restrictions.

## Supplementary Materials

The following supporting information can be downloaded at: https://www.mdpi.com/article/10.3390/children13101285/s1, Table S1: Omnibus test results of the multivariable analysis.

## Abbreviations

The following abbreviations are used in this manuscript:

HBoV	human bocavirus
PCR	polymerase chain reaction
RT-qPCR	reverse transcription quantitative polymerase chain reaction
PICU	pediatric intensive care unit
HFNC	high-flow nasal cannula
NLR	neutrophil-to-lymphocyte ratio
MLR	monocyte-to-lymphocyte ratio
PLR	platelet-to-lymphocyte ratio
SII	systemic immune-inflammation index
NPAR	neutrophil percentage-to-albumin ratio
WBC	white blood cell count
CRP	C-reactive protein
IQR	interquartile range
SD	standard deviation
CI	confidence interval
Ct	cycle threshold
PERCH	Pneumonia Etiology Research for Child Health

## References

[1] Madi N.M., Al-Adwani A. (2020). Human bocavirus (HBoV) in Kuwait: Molecular epidemiology and clinical outcome of the virus among patients with respiratory diseases. J. Med. Microbiol..

[2] Tan J., Huang Z., Tang W., Liu G., Li P., Wen J., Mo L., Yan C., Dang Z., Huang H. (2025). Human bocavirus-1 infection in hospitalized pediatric patients with acute respiratory tract infections. Microbiol. Spectr..

[3] Calvo C., García-García M.L., Pozo F., Carballo D., Martínez-Monteserín E., Casas I. (2016). Infections and coinfections by respiratory human bocavirus during eight seasons in hospitalized children. J. Med. Virol..

[4] Mijač M., Meštrović T., Ivković-Jureković I., Tot T., Vraneš J., Ljubin-Sternak S. (2024). The role of quantitative PCR in evaluating the clinical significance of human bocavirus detection in children. Viruses.

[5] Martin E.T., Kuypers J., McRoberts J.P., Englund J.A., Zerr D.M. (2015). Human bocavirus 1 primary infection and shedding in infants. J. Infect. Dis..

[6] Nascimento-Carvalho A.C., Vilas-Boas A.-L., Fontoura M.-S.H., Xu M., Vuorinen T., Söderlund-Venermo M., Ruuskanen O., Nascimento-Carvalho C.M., PNEUMOPAC-Efficacy Study Group (2018). Serologically diagnosed acute human bocavirus 1 infection in childhood community-acquired pneumonia. Pediatr. Pulmonol..

[7] Don M., Söderlund-Venermo M., Valent F., Lahtinen A., Hedman L., Canciani M., Hedman K., Korppi M. (2010). Serologically verified human bocavirus pneumonia in children. Pediatr. Pulmonol..

[8] Schlaberg R., Ampofo K., Tardif K.D., Stockmann C., Simmon K.E., Hymas W., Flygare S., Kennedy B., Blaschke A., Eilbeck K. (2017). Human bocavirus capsid messenger RNA detection in children with pneumonia. J. Infect. Dis..

[9] Caporizzi A., Ravidà F., Barneschi S., Moriondo M., Nieddu F., Boscia S., Stinco M., Ricci S., Trapani S. (2024). Analysis of a cohort of 165 pediatric patients with human bocavirus infection and comparison between mono-infection and respiratory co-infections: A retrospective study. Pathogens.

[10] Ademhan Tural D., Yalcin E., Emiralioglu N., Ozsezen B., Alp A., Sunman B., Gozmen O., Dogru D., Ozcelik U., Kiper N. (2022). Human bocavirus and human metapneumovirus in children with lower respiratory tract infections: Effects on clinical, microbiological features and disease severity. Pediatr. Int..

[11] Sun H., Sun J., Ji W., Hao C., Yan Y., Chen Z., Wang Y. (2019). Impact of RSV coinfection on human bocavirus in children with acute respiratory infections. J. Trop. Pediatr..

[12] Calvo C., García-García M.L., Pozo F., Paula G., Molinero M., Calderón A., González-Esguevillas M., Casas I. (2015). Respiratory syncytial virus coinfections with rhinovirus and human bocavirus in hospitalized children. Medicine.

[13] Okuyan O., Elgormus Y., Dumur S., Sayili U., Uzun H. (2023). New generation of systemic inflammatory markers for respiratory syncytial virus infection in children. Viruses.

[14] Erdede Ö., Sarı E., Külcü N.U., Yamanel R.G.S. (2023). Neutrophil-to-lymphocyte ratio and the systemic immune-inflammation index: Biomarkers in infants with bronchiolitis: A cross-sectional study. Jpn. J. Infect. Dis..

[15] Kızılsoy Ö.F., Korkmaz M.F., Şenkan G.E., Bozdemir Ş.E., Korkmaz M. (2024). Relationship between the systemic immune-inflammatory index and the severity of acute bronchiolitis in children. Lab. Med..

[16] Burrack N., Adar A., Goldbart A., Weissmann S., Cohen B., Hazan I., Horev A., Golan-Tripto I. (2023). Monocyte and neutrophil to lymphocyte ratios in hospitalized children with RSV bronchiolitis. Pediatr. Pulmonol..

[17] Matera L., Nenna R., Frassanito A., Petrarca L., Mancino E., Rizzo V., Di Mattia G., La Regina D.P., Pierangeli A., Midulla F. (2022). Low lymphocyte count: A clinical severity marker in infants with bronchiolitis. Pediatr. Pulmonol..

[18] Guo X., Luo H., Song Y., Xiao L., Xu X., Guo Y. (2025). Application value of systemic immune-inflammation index in predicting severe Mycoplasma pneumoniae pneumonia. PLoS ONE.

[19] Wang S., Wan Y., Zhang W. (2024). The clinical value of systemic immune inflammation index (SII) in predicting the severity of hospitalized children with Mycoplasma pneumoniae pneumonia: A retrospective study. Int. J. Gen. Med..

[20] Kalaycı F., Çelebier K. (2025). Differential laboratory findings of common respiratory viruses in hospitalized children: A retrospective study. Turk. J. Pediatr. Dis..

[21] Harris M., Clark J., Coote N., Fletcher P., Harnden A., McKean M., Thomson A., British Thoracic Society Standards of Care Committee (2011). British Thoracic Society guidelines for the management of community acquired pneumonia in children: Update 2011. Thorax.

[22] Fancourt N., Deloria Knoll M., Barger-Kamate B., de Campo J., de Campo M., Diallo M., Ebruke B.E., Feikin D.R., Gleeson F., Gong W. (2017). Standardized interpretation of chest radiographs in cases of pediatric pneumonia from the PERCH study. Clin. Infect. Dis..

[23] Moesker F.M., van Kampen J.J.A., van der Eijk A.A., van Rossum A.M.C., de Hoog M., Schutten M., Smits S.L., Bodewes R., Osterhaus A.D.M.E., Fraaij P.L.A. (2015). Human bocavirus infection as a cause of severe acute respiratory tract infection in children. Clin. Microbiol. Infect..

[24] Petrarca L., Nenna R., Frassanito A., Pierangeli A., Di Mattia G., Scagnolari C., Midulla F. (2020). Human bocavirus in children hospitalized for acute respiratory tract infection in Rome. World J. Pediatr..

[25] Oldhoff E., Bennet R., Eriksson M., Allander T. (2023). Human bocavirus 1 epidemiology in children in relation to virus load and codetection. Acta Paediatr..

[26] Chen Z.R., Mize M., Wang Y.Q., Yan Y.D., Zhu C.H., Wang Y., Ji W. (2014). Clinical and epidemiological profiles of lower respiratory tract infection in hospitalized children due to human bocavirus in a subtropical area of China. J. Med. Virol..

[27] Tang X., Dai G., Wang T., Sun H., Jiang W., Chen Z., Yan Y. (2023). Comparison of the clinical features of human bocavirus and metapneumovirus lower respiratory tract infections in hospitalized children in Suzhou, China. Front. Pediatr..

[28] Trapani S., Caporizzi A., Ricci S., Indolfi G. (2023). Human bocavirus in childhood: A true respiratory pathogen or a “passenger” virus? A comprehensive review. Microorganisms.

[29] Martin E.T., Fairchok M.P., Kuypers J., Magaret A., Zerr D.M., Wald A., Englund J.A. (2010). Frequent and prolonged shedding of bocavirus in young children attending daycare. J. Infect. Dis..

[30] De R., Zhang K.-X., Wang F., Zhou Y.-T., Sun Y., Chen D.-M., Zhu R.-N., Guo Q., Liu S., Qu D. (2022). Human bocavirus 1 is a genuine pathogen for acute respiratory tract infection in pediatric patients determined by nucleic acid, antigen, and serology tests. Front. Microbiol..

[31] Zhou L., Zheng S., Xiao Q., Ren L., Xie X., Luo J., Wang L., Huang A., Liu W., Liu E. (2014). Single detection of human bocavirus 1 with a high viral load in severe respiratory tract infections in previously healthy children. BMC Infect. Dis..

[32] Han B., Li X., Zhang L., Zhang H., Wang B. (2025). Clinical value of C-reactive protein to albumin ratio, aspartate aminotransferase, and platelet-to-lymphocyte ratio in predicting the severity of community-acquired pneumonia in children. Front. Pediatr..

[33] Elmeazawy R., Ayoub D., Morad L.M., El-Moazen A.M.F. (2024). Role of systemic immune-inflammatory index and systemic inflammatory response index in predicting the diagnosis of necrotizing pneumonia in children. BMC Pediatr..

[34] Ivaska L.E., Silvoniemi A., Palomares O., Turunen R., Waris M., Mikola E., Puhakka T., Söderlund-Venermo M., Akdis M., Akdis C.A. (2021). Persistent human bocavirus 1 infection and tonsillar immune responses. Clin. Transl. Allergy.

