# Supplementary material for: Early Hemogram-Derived Inflammatory Indices Do Not Independently Predict Hospital Stay in Children with Human Bocavirus Detected by Polymerase Chain Reaction
Source: Children (Basel). 2026 Sep 22;13(10):1285. doi: 10.3390/children13101285 (PMC13703595; doi:10.3390/children13101285)
Supplement: Supplementary file 1 [file children-13-01285-s001.zip › children-4560585-supplementary.pdf]

Supplementary Table S1. Omnibus test results of the multivariable analysis

	NPAR		SII		NLR		PLR	
	X^2	p	X^2	p	X^2	p	X^2	p
**Tachycardia**	6.14	0.013	5.51	0.019	5.65	0.02	5.68	0.017
**Neutrophil percentage**	5.74	0.017	11.42	0.001	17.35	0.000	4.01	0.045
**Neurological disease**	2.34	0.126	2.40	0.121	1.85	0.174	2.70	0.101
**Inborn Metabolic Disease**	1.71	0.191	1.38	0.240	1.44	0.230	1.82	0.177
**Cardiac disease**	0.03	0.860	0.02	0.900	0.00	0.995	0.08	0.777
**Index (p)**	1.79	0.181	1.07	0.300	4.49	0.034	1.09	0.296
**q (After BH-FDR)**		0.300		0.300		0.136		0.300

BH-FDR: Benjamini-Hochberg- false Discovery Correction
